# Comparison of Three Serological Methods for the Epidemiological Investigation of TBE in Dogs

**DOI:** 10.3390/microorganisms9020399

**Published:** 2021-02-15

**Authors:** Philipp Girl, Maja Haut, Sandra Riederer, Martin Pfeffer, Gerhard Dobler

**Affiliations:** 1 Bundeswehr Institute of Microbiology, D-80937 Munich, Germany; philippgirl@bundeswehr.org; 2 German Centre for Infection Research (DZIF), Partner Site Munich, D-80937 Munich, Germany; 3 Institute of Animal Hygiene and Veterinary Public Health, Faculty of Veterinary Medicine, University of Leipzig, D-04103 Leipzig, Germany; maja.haut@vetmed.uni-leipzig.de (M.H.); pfeffer@vetmed.uni-leipzig.de (M.P.); 4 Kleintierpraxis Dr. Christian Cronenberg, D-93173 Wenzenbach, Germany; sandra.riederer.bhw@gmail.com

**Keywords:** TBEV, seroprevalence, dog, ELISA, IIFA, micro-NT

## Abstract

Tick-borne encephalitis (TBE) virus is an emerging pathogen that causes severe infections in humans. Infection risk areas are mostly defined based on the incidence of human cases, a method which does not work well in areas with sporadic TBE cases. Thus, sentinel animals may help to better estimate the existing risk. Serological tests should be thoroughly evaluated for this purpose. Here, we tested three test formats to assess the use of dogs as sentinel animals. A total of 208 dog sera from a known endemic area in Southern Germany were tested in an All-Species-ELISA and indirect immunofluorescence assays (IIFA), according to the manufacturer’s instructions. Sensitivity and specificity for both were determined in comparison to the micro-neutralization test (NT) results. Of all 208 samples, 22.1% tested positive in the micro-NT. A total of 18.3% of the samples showed characteristic fluorescence in the IIFA and were, thus, judged positive. In comparison to the micro-NT, a sensitivity of 78.3% and a specificity of 98.8% was obtained. In the ELISA, 19.2% of samples tested positive, with a sensitivity of 84.8% and a specificity of 99.4%. The ELISA is a highly specific test for TBE-antibody detection in dogs and should be well suited for acute diagnostics. However, due to deficits in sensitivity, it cannot replace the NT, at least for epidemiological studies. With even lower specificity and sensitivity, the same applies to IIFA.

## 1. Introduction

Tick-borne encephalitis virus (TBEV) is an emerging tick-borne viral pathogen which causes severe, generalized infections involving the central nervous system (CNS) [1,2]. It is endemic in large parts of Europe and Asia [3,4,5] and with approximately 10,000–12,000 human cases per year, it is the most important vector-borne viral infection in Europe [6,7]. TBEV is a member of the family Flaviviridae, genus Flavivirus, and comprises three genetic subtypes: Western (TBEV-EU), Far Eastern (TBEV-FE), and Siberian (TBEV-Sib), with at least another four proposed subtypes (two Baikalian, Himalayan, Obskaya) [8]. The virus is transmitted by ticks of the genus Ixodes; in Germany and central Europe (TBEV-EU) this mainly occurs via *Ixodes ricinus*, while the Siberian and Far Eastern subtypes are mainly transmitted by *Ixodes persulcatus* [3,9,10]. In humans, symptoms caused by TBEV-EU often occur in a biphasic form, with unspecific flu-like symptoms in the first phase, which is followed by an asymptomatic period [3,5,11]. The second phase occurs in one-third of patients, characterized by high fever and increasing neurological disorders, ranging from photophobia, headache and vomiting, to tremor, reduced consciousness, cognitive deficits, paresis and in rare cases, death [3,5,11]. So far, risk areas in Germany and many other European countries are defined by the incidence of human cases [12,13]. However, rising vaccination rates may lead to a decrease in clinical human Tick-borne encephalitis (TBE) disease (e.g., Austria). In addition, human exposure to ticks can also vary greatly depending on the region, the season and year. All these aspects have a great influence on the current method of assessing risk areas and could lead to incorrect classification. [12,14]. Various animal species are also susceptible to tick-borne encephalitis (TBE) infection [15,16,17,18]. Unlike humans, animals rarely show clinical symptoms, but some cases have been described in horses, monkeys, sheep and dogs [19,20,21,22]. Despite occasional high seroprevalence rates, clinical manifestations of TBE are rare in dogs, even after experimental infection [23]. However, if symptoms develop, the disease often takes a severe course with up to 50% of cases being fatal [19,22]. Due to their smaller body size and their exploratory behavior on the ground, dogs have a 50 to 100 times higher risk of coming into contact with TBEV-infected ticks compared to humans [22]. It is, therefore, not surprising that in endemic areas the seroprevalence rate in dogs can be as high as 30–40% [24,25,26]. The close relationship to humans as companion animals make dogs a suitable sentinel species for surveillance and may indicate new emergence of TBE risk areas before the first appearance of human cases [24,27]. For the diagnosis of TBE infection in dogs, as in humans, serological test systems for the detection of TBE-specific antibodies are the method of choice [28]. While methods such as enzyme linked immunosorbent assays (ELISA) or indirect immunofluorescence assays (IIFA) may exhibit considerable cross reactions with other flaviviruses virus, neutralization test (NT) is considered the most specific serological assay [29]. Studies on the sensitivity and specificity of the detection of TBE antibodies in dogs are non-existent. Therefore, the aim of this study was to investigate the antibody prevalence in a dog population, in a well-known TBE-endemic region in south-eastern Germany and to compare the suitability of a commercially available ELISA, a modified IIFA and an in-house NT for epidemiological studies in dogs.

## 2. Materials and Methods

### 2.1. Samples

A total of 208 dog serum samples were included in the study, taken in a veterinary practice for pets between 2018 and 2019. Samples were drawn from the Vena saphena lateralis of clinically healthy dogs that came from a known TBE risk area. Either residual sera were used, or extra sera were taken for this study (Government of Lower Franconia permit AZ 2-673) and samples were anonymized for the testing. Sera were stored at −20 °C until use and at 4 °C during use in assays. Written consent of the dog owners was given for each individual dog. In addition, a questionnaire was completed in which relevant aspects such as age, travel history, place of residence and vaccination status of the dog were asked.

### 2.2. Micro-Neutralisation Test (micro-NT)

NT was conducted as a micro-NT according to standard procedure, as described before [30,31,32]. In brief, TBEV (strain Neudörfl) was cultured in A549 cells. Virus stocks (50 tissue culture infection dose (TCID)/50 µL) were prepared and stored at −80 °C until further use. The micro-NTs were performed in 96-well cell culture plates (Greiner bio-one, Frickenhausen, Germany). After complement-inactivation at 56 °C for 30 min, serum samples were tested in duplicate, diluted in Minimal Essential Medium (MEM, plus Non-Essential Amino Acids Solution and Antibiotic-Antimycotic Solution; all Invitrogen, ThermoFisher Scientific, Darmstadt, Germany) in dilutions of 1:10 to 1:1280. On each 96-well plate a defined positive and negative control was added together with a cell control and a virus back-titration. A total of 50 TCID of virus stock was added to the respective serum dilutions and incubated for one hour at 37 °C. Subsequently, 10^4^ cells (A549) were added per well and incubated for five days at 37 °C (5% CO_2_). The supernatants were then discarded and the 96-well plates were fixed in 13% formalin/PBS, stained with crystal violet (0.1%) and titers visually determined. The antibody titer corresponded to the highest serum dilution showing complete inhibition of cytopathic effect (CPE) in both wells were reported. Thus, the samples were classified as either “NT negative” (titer < 1:10) or “NT positive” (titer ≥1:10), with the highest readable titer being ≥ 1:1280.

### 2.3. IIFA

The IIFA (FSME-Viren (TBEV), Euroimmun AG, Luebeck, Germany) was performed according to the manufacturer’s instructions with the adaptation for the examination of dog samples instead of human samples. For this purpose, the supplied human-specific conjugate was replaced with an anti-dog conjugate (Abcam, Cambridge, UK) and used in a 1:20 dilution (pre-determined). Serum samples were diluted 1:10 and screened for TBEV-specific antibodies. The tests were read using a fluorescence microscope (Leica DM 5000B, Wetzlar Germany), independently by two experienced people and classified as either “positive” (characteristic cytoplasmic fluorescence visible, no fluorescence in control field) or “negative” (no characteristic fluorescence visible, no fluorescence in control field/uncharacteristic fluorescence in positive and control field), as suggested by the manufacturers. In cases of un-characteristic fluorescence in positive and control field samples, these were also considered as being negative.

### 2.4. All-Species ELISA

Immunozym FSME IgG All-Species ELISA (PROGEN Biotechnik GmbH, Heidelberg, Germany) was performed according to the manufacturer’s instructions. A Tecan Sunrise (Tecan, Männedorf, Switzerland) was used for photometric reading. The measured optical density was converted into Vienna units (VIEU/mL) based on the calibrator values. Samples with less than 63 VIEU/mL were evaluated as negative for TBEV-specific IgG antibodies, samples with 63 to 126 VIEU/mL as borderline and samples with more than 126 VIEU/mL as positive.

### 2.5. Statistical Analysis

Correlation between NT titers and ELISA (VIEU/mL) was evaluated using Pearson regression analysis (95% confidence interval). Statistical analyses were performed using GraphPad Prism 8 for Windows (GraphPad Software, San Diego, CA, USA).

## 3. Results

### 3.1. Detection of TBEV Neutralizing Antibodies by Micro-NT

**Figure 1:**
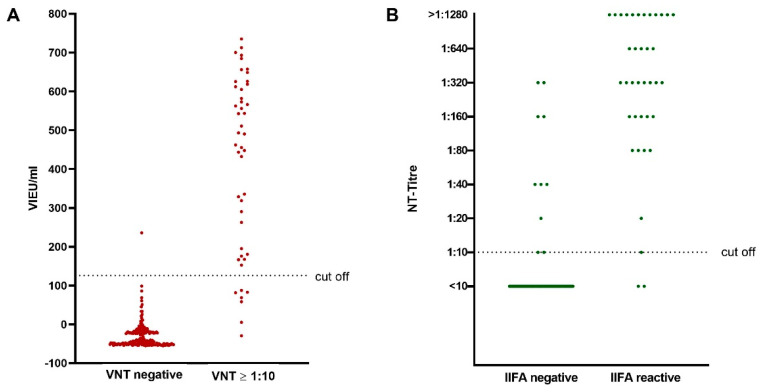
Distribution of (**A**) Vienna units (VIEU)/mL determined by ELISA within negative and positive neutralization test (NT) results and (**B**) of NT-titers within negative and positive IIFA results.

Of all 208 serum samples, 22.1% (46/208) tested positive (titer ≥ 1:10) for TBEV-specific antibodies, while 77.9% (162/208) showed no reactivity (titer < 10) in the micro-NT. Of the positive tested samples, eight (17.4%) had a titer between 1:10 and 1:40, 21 (45.7%) had a titer between 1:80 and 1:320 and 17 (37.0%) had a titer of 1:640 or greater (Table 1 and Figure 1A,B).

### 3.2. Detection of Antibodies with the IIFA

A total of 38 of 208 (18.3%) samples showed characteristic fluorescence in the IIFA and were classified as positive for TBEV-specific antibodies, while 170 samples (81.7%) tested negative. Two IIFA-positive samples were found to be negative in the NT, while eight sera showing a positive reaction in NT were non-reactive in IIFA. A total of 36 out of the 46 micro-NT-positive sera were also positive in the IIFA, corresponding to a sensitivity of 78.3%. On the other hand, IIFA confirmed 160 of the 162 micro-NT-negative samples, indicating a specificity of 98.8% (Table 1 and Figure 1B).

### 3.3. Detection of Antibodies by All-Species-ELISA

In the All-species-ELISA, 40 sera (19.2%) tested positive for TBEV-specific antibodies. A total of 39 out of 46 micro-NT-positive samples were correctly identified as positive, resulting in a sensitivity of 84.8%. A total of 161 of the 162 micro-NT-negative samples were correctly identified in the ELISA, thus showing a specificity of 99.4%. One ELISA-positive serum showed no reaction in NT. In comparison with the IIFA, 36 out of the 40 ELISA-positive sera (90%) were confirmed, while two (1.2%) of the ELISA-negative samples showed a positive reaction in the IIFA (Table 1 and Figure 1A).

## 4. Discussion

TBEV transmission to animals is an important topic, especially since several animal species represent excellent sentinels for human TBE risk assessment [12,33,34,35]. A number of seroprevalence studies (reviewed in [19,22]) have shown that TBEV antibodies can be found in dogs from endemic areas. The antibody prevalence rates vary depending on the region and on the health status of the tested dogs. In a number of studies in known TBE-endemic areas Germany and Austria, the antibody prevalence rate has been determined to range between 10 and 30%. In one study in Germany, the prevalence rate in healthy dogs was 31%, while the prevalence rate in dogs with neurological symptoms was 53% [36]. The antibody prevalence rate of 22.1% found in the current study lies well in the range of earlier studies and confirms that the dogs were coming from an endemic area in south-western Germany.

Sensitive serological assays that reliably and specifically detect antibodies against TBEV form the basis of corresponding epidemiological investigations. In this context, the NT assay is still considered the most specific serological test method [29,37]. At the same time, the NT also has some disadvantages. Working with TBE viruses requires a BSL3 laboratory with appropriately trained personnel, which limits the test procedure to specialized laboratories and therefore makes it expensive. In addition, it takes a few days for the results to be obtained. Therefore, it is obviously beneficial to replace NT wherever possible, with simpler, faster and cheaper test methods such as ELISA or IIFA. The Immunozym FSME IgG All-Species-ELISA used in this study has already been frequently used in other studies [24,37,38,39]. In most cases, ELISA is used as a screening test and NT is performed, if at all, only to confirm ELISA-positive sera as a second step [40,41]. Few studies have investigated the performance of the Immunozym FSME IgG All-Species-ELISA in terms of its sensitivity and specificity. Corresponding data on dogs are so far available from one study in Denmark [25]. In this study, however, only ELISA-positive sera were used for confirmation by NT. Therefore, sensitivity and specificity could only be calculated under the premise that all ELISA-negative results would also have tested negative in the NT. 

The objective of this study was to compare the performance of the only commercially available ELISA assay for animals (Immunozym FSME IgG All-Species-ELISA) in testing dog samples with a commercially available IIFA (FSME-Viren (TBEV), Euroimmun) using an in-house NT as reference test. The NT is considered the most specific serological test, but not necessarily the most sensitive. Accordingly, the significantly poorer sensitivity of ELISA (84.8%) and IIIFA (78.3%) in this comparison was surprising. However, the sensitivity of the ELISA was lower in other animal species studied, ranging from only 32% in goats to 42.3% in foxes and 63.6% in roe deer [32,35,38]. This lower sensitivity of the ELISA in the other studies could be due to the method of sample collection, at least for foxes and roe deer: In both studies, the samples were obtained post-mortem from hunted animals, which may have altered the quality of the sera compared with samples from live animals. Conversely, the specificity rates of IIFA (98.8%) and ELISA (99.4%) were comparatively high, almost reaching that of NT. This high specificity of the ELISA is consistent with previous studies that have used Immunozym ELISA in the analysis of goats and foxes, where it achieved specificities of 100 and 98.9%, respectively [32,37]. Maybe dogs produce higher amounts of TBE antibodies after infection, or many of the dogs may be exposed and infected several times with TBE virus causing higher antibody titers than other animals, or perhaps TBEV antibodies in dogs persist for a longer period of time compared to in other animal species. So far, we are not aware of any studies on the dynamics of TBEV antibodies in dogs.

Sera tested as reactive in IIFA and/or ELISA that could not be confirmed in NT were scored as “false positive”. However, it is possible that IIFA and ELISA actually detect specific anti-FSME antibodies in such sera, which do not exhibit a neutralizing capacity and are, therefore, not detected in the NT. Another possibility could be the cross-reactivity with other circulating flaviviruses. So far in the studied region, there is no evidence of the circulation of, e.g., West Nile virus. It was also ensured that the dogs from the study had not previously been vaccinated with a (human) TBE vaccine, which would have led to false positive results. From human diagnostics we know that IgG may react in an unspecific way in patients with rheumatic diseases. As there was no information about pre-existing diseases in the dogs, this possibility cannot be completely excluded. In our study, however, only two sera (ID 105 and ID 178) were affected by this definition, so the negative impact on the specificity of ELISA and IIFA could be considered minor. It must also be considered that the data collected are from dogs in an endemic area. Therefore, the positive predictive value is likely to be higher than in a non-endemic area, due to a higher prevalence. Consequently, the number of false positive IIFA/ELISA results in a non-endemic area could be significantly higher than in this study. This might also explain why the correlation between ELISA and NT results in this study is significantly stronger than in the study from Denmark, where dogs from non-endemic areas were also included [25]. In the Danish study, 30.4% of the samples were ELISA positive but only 4.8% were also positive in the NT, whereas in this study 19.2% of the samples were positive in the ELISA and 22.1% in the NT. The Danish authors cite changes in the antigen profile during transovarial transmission in the tick population as a possible cause of ELISA-positive sera lacking neutralising activity, which could have implications especially in areas with only sporadic TBE cases [25,42]. However, another difference between the two studies results from the use of different ELISAs: The ELISA of the Danish study exhibited a significantly higher sensitivity (100 vs. 84.8%) with a lower specificity (57.6 vs. 99.4%).

In our evaluation, we also conservatively considered borderline ELISA results as negative. On the other hand, if borderline results were considered positive, the sensitivity of the ELISA would increase significantly (93.5 instead of 84.8%), but at the expense of specificity (97.5 instead of 99.4%). Interestingly, for the ELISA borderline sera, even the level of VIEU/mL values could not assist in deciding whether the respective sera were better considered negative or positive. Of the seven ELISA borderline sera, four tested positive in micro-NT (mean VIEU/mL value: 80.12) and three tested negative (mean VIEU/mL value: 84.47). In terms of sensitivity and specificity, the Immunozym ELISA seems to have been designed for acute diagnostics rather than for epidemiological investigations. In particular, the two-step test set-up frequently used so far, with an ELISA as the screening test and a downstream NT to confirm positive results, does not appear to be effective in view of the poorer sensitivity of ELISA compared with NT. In particular, sera with declining antibody titers from animals with TBEV infection that occurred in the distant past, or from animals right at the onset of the disease, could be missed using ELISA as a screening test. The same applies to the IIFA tested here, which was again inferior to the ELISA in terms of sensitivity and specificity.

In summary, the Immunozym FSME IgG All-Species-ELISA is a highly specific test used for the detection of antibodies against TBEV in dogs and should be well suited for acute diagnostics. However, due to deficiencies in sensitivity, it cannot be recommended for use as a screening test or even as a sole test for epidemiological purposes. Although the Immunozym ELISA performed significantly better in our study in dogs compared with previous studies in other animal species, it cannot replace the NT assay, at least for epidemiological studies. With even lower specificity and, above all, sensitivity, the same applies to IIFA.

## Figures and Tables

**Table 1:** Comparison of sensitivity and specificity of indirect immunofluorescence assays (IIFA) and ELISA. A total of 78.3% (36/46) of all micro-neutralization test (NT)-positive sera were confirmed by IIFA, while ELISA correctly identified 84.8% (39/46) as positive. Among the NT-negative samples, IIFA and ELISA correctly identified 98.8 (160/162) and 99.4% (161/162) as negative, respectively. Both the IIFA and ELISA results had a statistically significant correlation with the NT result (*p* < 0.0001 Fisher’s exact test).

Serum Samples		IIFA		All-Species-ELISA	
		Positive	Negative	Positive	Negative
Total	208	38	170	40	168
micro-NT positive	46	36	10	39	7
micro-NT negative	162	2	160	1	161
Sensitivity (%)		78.3		84.8	
Specificity (%)		98.8		99.4	

## Data Availability

The data presented in this study are available on request from the corresponding author. The data are not publicly available due to privacy restrictions.
